# Pathological Notes from the Autopsy Records of the Columbia Veterinary College

**Published:** 1880-01

**Authors:** 


### ABSTRACT AND SUMMARY.

### PATHOLOGICAL NOTES FROM THE AUTOPSY RECORDS OF THE COLUMBIA VETERINARY COLLEGE.

1. Rupture of Urinary Bladder in a Lioness.—A full-grown lioness was received from the Director of the Zoölogical Department of the Central Park, with the following history: Five days before death she was delivered of three cubs, and nothing unusual was noticed (though naturally a searching examination was not made); on the morning of the fifth day *post partum* she was found dead in the cage. Autopsy eight (?) hours *post mortem:* Cadaveric rigidity well marked, abdominal cavity distended; on opening, a large amount of turbid serum escaped. The serous surfaces of the intestine, mesentery and pelvic organs of a rosy hue, with pronounced arterial injection. Liver, kidney, spleen and alimentary tracts healthy, but all more or less hyperæmic; uterus in normal involution. There were *four* placentar zones, of which one was ill-marked (three cubs!). On the posterior wall of the bladder, in the middle line and between the vertex and ureteral mouths, there was was a large transverse rent, with rather regular edges.

The mucous membrane of the bladder was found thickened and ulcerated, especially at and near the rent. Several nodules, some pale, others with apopleptic clots in their softened centres, were scattered throughout the more intact portions of the mucous membrane. The bladder was not contracted.

Lungs, the seat of marked hypostasis, several tubercular (caseous) nodules, with a zone of reactive inflammation in the posterior lobes of both lungs. Pericardium distended by clear serum, heart normal, cerebro-spinal meninges congested, nerve centres otherwise healthy.

Anatomico pathological diagnosis: Acute peritonitis following rupture of bladder, produced by over-distention, and ultimately due to ulceration of the same from the breaking down of caseous nodules. Cheesy pneumonia as a collateral lesion, in no immediate relation to the cause of death, which was presumably the shock of the first stage of peritonitis.

2. Tumor of Dura Mater in Horse.—See Article II. in this number of the Archives.

3. Acute Suppurative Peritonitis in a Sea-lioness.—A sea-lioness (*Zalophus gillespici*), under the charge of Mr. Conklin, refused food, and several days subsequently developed diarrhœa, the hair assuming that peculiar ruffled appearance which in these animals is almost always a presage of death. No medicines could be administered. On the thirteenth or fourteenth day of the illness the animal began to feed again and the passages became less frequent; on or about the twentieth day the dead body arrived at the College without further history. The cerebro-spinal meninges were found hyperaemic, heart and lungs healthy. On opening the abdomen, a moderate quantity of slightly turbid serum, with numerous flocculi of lymph, escaped. Long strips of lymph were found among the intricate intestinal coils, and extensive pseudo-membranes of the same material, and a whitish-yellow color, covered the convex surface of the liver, the spleen, and diaphragm. On introducing the hand into the pelvic cavity, a pint and a half of creamy yellow pus escaped from the latter.

The intestinal tract, throughout its extent, presented maceration of the epithelium, irregularly excavated ulcerations, and other signs of chronic enteritis. On account of lack of assistance, and the presence of dissecting wounds, the examination was discontinued. But that, either by extension through contiguity of inflammation, or by perforative ulceration, the enteritis led to the peritonitis, was the conclusion arrived at.

4. Embolic Pyaemia of Horse.—A car-horse in the hospital department of the Third Avenue R. R. stables (under the charge of Mr. Sulay, the house surgeon), with extensive suppurative and necrotic destruction of the tissues of the right hind foot, and having, for a month, presented rigors, rises in temperature, and other febrile symptoms, was destroyed by order of the house-surgeon, and immediately autopsied in presence of several students and the Professor of Anatomy of the college. Nothing very noticeable was found except in the lungs; these contained several hundred large and small abscesses, ranging from the size of a pea to that of an orange. Most of these were diffused at their boundary, and the neighboring tissue was hyperaemic and oedematous. In some the pus was ichorous, but in most benign. Traces of phlebitis were found in the affected leg. The pleura was dull in its reflection over the larger abscesses (which protruded above the normal pulmonary contour), but otherwise nornal.

5. Catarrhal Pneumonia in a Hippopotamus.—A hippopotamus two years old, the property of "Barnum's Menagerie," and at the time on exhibition at the N. Y. Aquarium, was, through the carelessness of some carpenters, exposed to the cold night-air, in November, 1877. It refused food, and died three nights after with signs of aggravated dispnoea. On autopsy, the organs were all found more or less congested, and the right side of the heart filled, like the great veins, with dark coagulated blood. The lungs were in a state of red hepatization, tore easily under the finger, and sank in water.

A remarkable, to us not yet clear, condition was found in the brain. Right over the valve of Vieussens, proceeding from the vascular tunic, and involving in its substance one of the trochlearis nerves, was an irregular mass of true bone, measuring in every direction about three millimeters, and but loosely attached to the fibrous tentorium ; falx ceribri hardly to be identified.

6. Parasites of a Monster Sunfish.—A short sunfish (*Orthagoriscus mola*), found in an exhausted condition off Seabright, N. J., and which died an hour after its arrival in the N. Y. Aquarium, was found to be perfectly riddled by the long bodies of an undetermined crustacean, which, penetrating the shagreen-like skin to the depth of from three to six inches, could hardly be detached by force. In addition, several Caliguli and other Amphipoda were found attached under the fins, while large shield-shaped monostomate worms covered the operculum so closely that at first they were taken for the natural surface. Taenia had crawled out of its mouth, some of which measured over twenty feet in length, and which length was possessed by four individuals. Besides, there were several hundred ascarides. The most remarkable feature was, that a score of an undetermined species of leech were attached to the lateral margin of the tapeworm links. The specimens in the museum of the Columbia Veterinary College are awaiting identification. The leech last referred to was certainly the parasite of a parasite. The sunfish weighed 150 pounds.

7. CYSTICERCUS OF BRAIN IN ANTELOPE.—A SouthAfrican antelope (*Cephalophus mergens*), which had an abscess in the submaxillary region, with formation of sinuses, and died, from gradual weakness, with stupor and loss of appetite, was found to have no other internal lesion except a single cysticercus in the anterior part of the right cerebral hemisphere. The surrounding cerebral substance was normal.

8. CYSTICERCI IN BRAIN OF BABOON.—A large baboon (*Cynocephalus babinis*), from the N. Y. Aquarium, was noticed to behave strangely, act as if blind, and to have become so stupid, that his cage being left open, he failed to take the opportunity of escaping, and acted cowardly when a stranger or another monkey approached him, although previously a bold and aggressive animal. On the autopsy, two cysticerci were found in the cerebral cortex; one in the anterior part of the angular gyrus, the other in the frontal lobe of the right hemisphere.

9. METASTATIC MENINGITIS IN A FOX.—An emaciated specimen of the red fox (*Vulpes Americanus*) was sent to the laboratory by Mr. Conklin. Nothing was discovered of any moment in the thoracic and abdominal viscera. An abscess was found involving the parotid gland, and extending under the temporal muscle, and directly touching the surface of the temporal bone, denuded here of periosteum. The bone itself appeared intact. On removing the calvarium, the dura was found tense upon the side of the abscess (left side), and a large amount of turbid serum escaped from the arachnoid space. The pia was deeply injected, and flocculi of coagulable lymph covered the frontal and uncinate gyri. The veins from the temporal bone to the pia (which have, strangely enough, not received a careful anatomical description as yet) were thrombotic. The animal had exhibited "fits" before death, but whether unilateral or not, or of what nature, could not be ascertained.

